# “Hopefully, I will never forget that again” – sensitizing medical students for drug safety by working on cases and simulating doctor-patient communication

**DOI:** 10.3205/zma001225

**Published:** 2019-03-15

**Authors:** Verena Kirsch, Wencke Johannsen, Christian Thrien, Stefan Herzig, Jan Matthes

**Affiliations:** 1 Universität zu Köln, Zentrum für Pharmakologie, Köln, Germany; 2 Universität zu Köln, Medizinische Fakultät, Studiendekanat, Kölner Interprofessionelles Skills Lab und Simulationszentrum (KISS), Köln, Germany; 3 Technische Hochschule Köln, Köln, Germany

**Keywords:** medical education, pharmacovigilance, clinical pharmacology, health communication, simulation patient

## Abstract

**Objective:** This project is part of the “PJ-STArT-Block”, a one-week course preparing 10^th^ semester medical students for their final practical year. The focus is on sensitizing students to aspects of medication safety by becoming aware of their skills and their deficits in terms of application and communication of pharmacological knowledge. The modules were evaluated regarding feasibility, acceptance and possible effects. Furthermore, the areas in which students see their pharmacological deficits or learning successes were gathered.

**Methods: **In simulated physician-patient conversations, the students are to identify drug-related problems such as medication errors, adverse drug events or interactions. Together with their fellow students and under medical or pharmaceutical moderation, they then have to find solutions for the identified problems and communicate these solutions to the patients. Based on paper cases, students practice, reflect, and discuss the research of reliable information about drugs and medication therapy. The written evaluation included the evaluation by school grades and the possibility of comments in free text. A content analysis of interviews with students at the beginning of the project aimed to identify areas of pharmacology in which they see their own deficits.

**Results: **Evaluation results including the free text comments indicate students’ acceptance of our pharmacology modules. According to this, the students realize the importance of aspects relevant for medication safety. The areas mentioned in 35 interviews in which students localize deficits, correspond to the topics that were intended when conceiving the modules and which are important for medication safety (e.g. interactions, adverse drug effects, dosages).

**Conclusion:** Implementation of context-based, application-oriented teaching formats as recently claimed for pharmacological education to improve the quality of prescriptions, is possible, as the Cologne example shows. The student evaluation turns out positively and indicates a critical self-reflection. The students identified various pharmacological deficits in themselves, which have since been confirmed and quantified in another study.

## Zusammenfassung

**Zielsetzung: **Das Projekt ist Teil des PJ-STArT-Blocks, einer einwöchigen Vorbereitung Medizinstudierender des 10. Semesters auf das Praktische Jahr. Im Vordergrund steht, Studierende für Aspekte der Arzneitherapiesicherheit zu sensibilisieren, indem sie sich ihrer Fähigkeiten bzw. Defizite bezüglich der Anwendung und Kommunikation pharmakologischer Kenntnisse bewusst werden. Die Module wurden mit Blick auf Durchführbarkeit, Akzeptanz und mögliche Effekte evaluiert. Des Weiteren wurde erhoben, in welchen Bereichen die Studierenden grundsätzlich pharmakologische Defizite bzw. Lernerfolge bei sich sehen.

**Methodik: **In simulierten Arzt-Patient-Gesprächen sollen die Studierenden arzneimittelbezogene Probleme wie Medikationsfehler, unerwünschte Arzneimittelwirkungen oder Interaktionen erkennen, gemeinsam mit Kommiliton/inn/en unter ärztlicher bzw. pharmazeutischer Moderation Lösungen für die vorliegenden Probleme finden und diese den Patient/inn/en kommunizieren. Anhand von Papierfällen üben, reflektieren und diskutieren die Studierenden die Recherche zuverlässiger Informationen zu Arzneimitteln und Arzneitherapie. Die Evaluation der Module erfolgte schriftlich anhand von Schulnoten und Freitextkommentaren. Eine inhaltsanalytische Auswertung von Interviews mit Studierenden zu Beginn des Projekts galt der Identifizierung von Bereichen der Pharmakologie, in denen sie eigene Defizite sehen.

**Ergebnisse: **Die Akzeptanz der Pharmakologie-Module kann angesichts der Evaluationsergebnisse inkl. Freitextkommentaren als gegeben angesehen werden. Die Studierenden erkennen demnach die Bedeutung von für die Arzneitherapiesicherheit relevanten Aspekten. Die in 35 Interviews genannten Bereiche, in denen Studierende Defizite lokalisieren, entsprechen den bei der Konzeption der Module intendierten, für die Patient/inn/ensicherheit bedeutsamen Themen (z.B. Interaktionen, unerwünschte Wirkungen, Dosierungen).

**Schlussfolgerung: **Das Kölner Beispiel zeigt, dass die erfolgreiche Umsetzung kontextbasierter, anwendungsorientierter Unterrichtsformate wie sie aktuell mit dem Ziel einer verbesserten Verordnungsqualität für die pharmakologische Ausbildung gefordert werden, möglich ist. Die studentische Evaluation fällt positiv aus und lässt eine kritische Selbstreflexion erkennen. Die Studierenden sehen verschiedene pharmakologische Defizite bei sich, die in einer weitergehenden Untersuchung bestätigt und quantifiziert wurden.

## 1. Introduction

### 1.1. Thematic background

An adverse drug reaction (ADR) is a drug effect that is harmful and unintended [1], [2]. According to the EU guideline 2001/83/EC, in the European Union not only reactions after intended use, but also medication errors as well as the misuse of medicinal products are considered to be ADR [http://eur-lex.europa.eu/legal-content/DE/TXT/?uri=celex%3A32001L0083].

According to the World Health Organization, 7-10% of patients in acute care suffer from ADR and about 10% of all hospitalizations are caused by an ADR [3]. Further studies in Germany and elsewhere show that ADR cause about 5% of hospital stays, whereby a majority can be classified as avoidable [3], [4], [5].

The German Ministry of Health’s plans of action to improve medication safety are important for avoiding ADR and medication errors in Germany. One topic area of the plans of action 2016-2019 is to raise awareness for avoidable risks in drug therapy [6], which should ideally begin during the education of the responsible occupational groups.

Due to their lack of experience, young professionals represent a group at substantial risk [7]: up to 10% of their prescriptions exhibit relevant errors [8], [9]. For prospective physicians, the prescription of drugs as well as the evaluation and, if necessary, adaptation of existing drug therapies are of essential importance for at least two reasons: firstly, they are allowed by law to prescribe as soon as they have their medical license, and secondly, they have to prescribe on their own responsibility at an early stage in their daily practice. However, in the final year of medical studies and in the first year of working life, there are still significant deficits in prescribing drugs [8], [10]. The lack of context-based, application-oriented teaching formats is an obvious weakness of pharmacological education throughout Europe [11]. The project presented here has been using exactly such formats for years to enable students to become aware of their own abilities or deficits regarding the use and communication of pharmacological knowledge immediately before the final practical year (“PJ”) e.g. by simulating daily hospital routines. The increase of awareness for important aspects of medication safety aimed at by the project should give rise to pay closer attention to drug-related problems in the future.

#### 1.2. Setting

##### 1.2.1. Reformed Medical Curriculum at the University of Cologne

After the approval of the 9^th^ amendment of the Medical Licensure Act (ÄApprO) in 2003, medical studies at the University of Cologne were reorganized and a new concept was implemented to impart medical knowledge as well as medical skills and abilities. Already in the pre-clinical part (four semesters), there are interdisciplinary courses, the so-called Kompetenzfelder (literally: fields of competence), that are supported or even managed by clinicians. In the clinical part (six semesters), the first twelve to fourteen weeks of a semester are mainly for discipline-based courses, Kompetenzfelder and skills trainings. In the last two to three weeks, examinations are held and students deepen their knowledge and skills in electives [12], [13].

##### 1.2.2. The PJ-STArT Block 

Since 2009, the one-week, mandatory PJ-STArT-Block (**S**chlüsselkompetenz-**T**raining und **A**nwendung in **r**ealitätsnahen **T**agesabläufen; translatable as: key-competence training and application in realistic daily routines) takes place for 10^th^ semester students. The course was implemented as part of the interdisciplinary, cross-faculty, innovative teaching project EISBÄR (**E**ntwicklung und **I**ntegration von **S**chlüsselkompetenzen des **B**erufsbildes von **Är**zt/inn/en; translatable as: development and integration of key competences of a physician’s job profile), funded by the Rectorate of the University of Cologne. Currently it is financed through means for quality improvement of the federal state of North Rhine-Westphalia. It consists of 18 different modules in which 24 students per week are confronted with both simulated patients and cross-case tasks. In addition to handling patient cases, daily morning and noon meetings, simulated works on the ward and several hours ward rounds complete the simulation of daily clinic routine. The modules are mainly taught in small groups of four students and moderated by lecturers from different departments. This is deliberately done also by non-medical staff, e.g. pharmacists. The modules conceived and moderated by the center of pharmacology, mainly focus on a safe medication therapy in order to sensitize students to the identification and prevention of medication errors and to enable them to become aware of their own relevant strengths and weaknesses.

## 2. Project description

The Center of Pharmacology at the University of Cologne is contributing to the PJ-STArT-Block with four modules. There are three case scenarios with simulated patients (S1-S1'', “Heart & Lung”) and the “Quality Circle for Pharmacotherapy” (S8), which deals with paper cases.

### 2.1. Description of the pharmacology modules

#### 2.1.1. Pharmacology modules with simulated patients (S1-S1'' “Heart & Lung”)

There are three different scenarios (see table 1 (Tab. 1), table 2 (Tab. 2) and table 3 (Tab. 3)). In each case four students complete a module at the same time. In every scenario, one student assumes the role of the physician, while his or her fellow students and the lecturer observe the conversation through a mirrored window. The procedure is always the following:

1. First meeting with the patient to take a detailed history. The student stops independently when all essential information seems to be gathered (10-15 min.).
2. Away from the patient, discussion of the gathered information with fellow students in the sense of a “meeting among colleagues”. The first aim is to identify or confirm and, if necessary, to prioritize the patient’s problems. Among other things, medication errors (cf. table 1 (Tab. 1), table 2 (Tab. 2) and table 3 (Tab. 3)) are to be discussed here. Eventually, it has to be decided how to proceed with the patient and what to convey to him or her immediately. The lecturer primarily is a moderator here, but can contribute further findings (e.g. laboratory values), subject-matter questions or comments if needed (15-20 min.).
3. The student continues the doctor-patient conversation and informs the patient about the result of the “discussion among colleagues”. At least the further procedure and the reasons for it should be discussed here. At this point at the latest, medication errors that have been identified should be addressed (10-15 minutes).
4. A final round of feedback with all participants is primarily related to doctor-patient interaction, but may also include technical aspects if these cannot remain uncommented (e.g. the handling of medication errors) (10-15 min.).

The written information on the respective patient is always introduced with the sentence “You are an assistant physician in our interdisciplinary admission ward”. The information says that the patient has been undergoing drug treatment by a general physician for three years for symptomatic heart failure (NYHA II) and for six months for COPD (°II-III). This is followed by information on acute symptoms:

“Mr/Mrs Reuther has been suffering from tachycardia and shortness of breath for 3-4 days”.

“Mr/Mrs Oppermann now comes to you in the admission ward and complains about pain and edema in the legs”.

“Mr/Mrs Steigmüller was admitted to the hospital yesterday for a tachyarrhythmia absoluta due to paroxysmal atrial fibrillation and was immediately anticoagulated by your colleague”.

The short patient descriptions are followed by two work orders:

*“1^st^ patient contact: Please have a conversation to take the symptom-based history, including drug history.*

*2^nd^ patient contact: Please convey the further procedure to the patient afterwards”.*

##### 2.1.2. Pharmacology module with paper cases (S8 “Pharmacotherapeutic quality circle”)

This one-hour module takes place with all 24 students of the respective PJ-STArT-Block week. Small groups of 3-6 students work on different paper cases, which deal with pharmacokinetic and pharmacodynamic drug properties. The students are supposed to identify and evaluate interactions and ADR and, if necessary, make suggestions on how to avoid them. The scenarios include:

- Interaction between ibuprofen and low-dose aspirin. Specific instructions for drug use (sequence and interval) should be given, taking into account differences in mechanism of action.
- Consideration of renal failure in dosing of cetirizine.
- Recognizing bronchial asthma as a restraint for migraine prophylaxis by beta-blockers.

The students have the possibility to do research on the internet using provided tablet PCs. The module primarily aims at students getting to know various possibilities of information procurement, assessing the quality of the sources used and discussing their suitability as a basis for therapy decisions. The paper cases processed in small groups are then presented to the entire group and discussed under the moderation of the lecturer. The focus is on the used and not used sources.

#### 2.2. Evaluation of the pharmacology modules

All modules of the PJ-STArT-Block are evaluated at the end of a week. The students evaluate each individual module, but also the course as a whole, anonymously and in writing (“Use school grades 1-5”). In addition, students are asked to submit free text comments, which have been analyzed exploratively here. The medical faculty’s evaluation was carried out online at the end of each semester via the local campus management system “uk online”, using a Likert scale from 1 to 5 (“overall I rate the course with the grade”).

#### 2.3. Student self-assessment

At the beginning of the project (winter semester 2009/2010 and summer semester 2010), 35 randomly selected 10^th^ semester students (12%) were interviewed in semi-structured, problem-oriented interviews. Among other things, this was to find out, in which areas of pharmacology they most likely see their learning successes during their previous studies or where they rather perceive deficits [14]. The results were used to examine the modules’ content alignment. On the last day of each PJ-STArT-Block week, two students from two small groups of four students each were randomly selected and asked about their willingness to participate in an immediate interview. Participation was voluntary. The only prerequisite was having passed the courses on basic (6^th^ semester) and clinical pharmacology (9^th^ semester). 75% of the interviewees were female. The introductory question was: “In which areas do you see your personal deficits in pharmacology?”. In problem-centred interviews, the interviewees’ experiences, perceptions and reflections on a specific topic are of primary interest, without any options two answer being given [15]. The transcripts of the audio recordings of the interviews were evaluated by inductive development of categories in a content analysis according to Mayring [16]. With reference to anchor examples the content of the material was summarized and presented in several levels of subcategories, considering the latent content. Content analysis always takes into account the context of the interview and of the interviewees. The categorisation was confirmed by the same person re-encoding three interviews (Cohens κ=0.9). Based upon the results, questionnaires were developed and used in a further study [17].

## 3. Results

### 3.1. Implementation of the modules

The lecturers were physicians or pharmacists. In light of the “typical” contents relevant to everyday practice (see table 1 (Tab. 1), table 2 (Tab. 2) and table 3 (Tab. 3)), supporting students was possible without an excessive preparation or even in-depth reading of relevant specialist literature being necessary. According to the experience of the lecturers, however, a medical or pharmaceutical qualification is required in order to ensure a targeted moderation of the discussion, which is conducted between the two "doctor-patient" contacts. All lecturers had taken part in a one and a half day didactic workshop “Interactive small group teaching” of the medical faculty in order to be prepared for the moderation of small groups, the use of activating methods and moderating feedback. New lecturers initially joined experienced colleagues, but were largely free in the individual organization of their own moderation. Usually the lecturers reported that it was a challenge that the modules had to be moderated continuously for up to seven weeks.

#### 3.2. Evaluation and acceptance of the modules

Irrespective of the change of lecturers over time, the written evaluation of the modules shows a consistently positive evaluation throughout the semesters. Between the summer semesters 2013 and 2017, the S1 modules were evaluated with an average grade of 1.8±0.1 (± standard deviation), the PJ-STArT-Block in total with a grade of 1.6±0.1 (mean values of the results of the semesters, based on a total of 875 and 838 responses, corresponding to a response rate of about two thirds). It should be noted that the PJ-STArT-Block has always been one of the best rated courses in our medical studies since its introduction. The “Quality Circle for Therapeutic Medicine” still performed well with 2.2±0.1 (N=699), although clearly worse, which was also observed in other PJ-STArT-Block modules without simulated patients.

Regarding the modules “Heart & Lung”, the open commentaries appreciated the two-times patient contact as well as the “*good exercise to identify complex connections”*. Comments such as *“drug interactions/ contraindications are always important and interesting”* or *“hopefully, I will never again forget to check the infusion pump again”* show that the importance of medication safety has been recognized. It has often been positively evaluated that the handling of mistakes is practiced. For example, it reads *“seeing how to convey errors is very helpful”* or *“practicing to apologize for errors is very reasonable”*. Furthermore, suggestions for improving education are being made, such as* “perhaps a small pharma repetition course [...] would be more helpful”* or *“would be better with a physical (e.g. auscultating)”*. Some feedback refers to the role of the individual knowledge level. While on the one hand the modules are described as *“tricky”*, on the other hand there are statements such as* “differences in students’ knowledge, therefore somewhat sluggish”* or “*patient case not particularly exciting”*.

The module “Pharmacotherapeutic quality circle” is appreciated by the students describing the *“dealing with media [as] positive”* and the setting as *“up-to-date”*. Again, the students notice the relation to drug safety (e.g. *“good exercise on interactions and adaptation in renal failure, etc.”* and* “have learned some important things about interactions through the distributed cases”*). Quotations such as *“cases of high clinical relevance”* and *“relevant to medical practice”* show the acceptance of this module. Here again the dependence on the level of knowledge is evident (e.g. *“use of the Rote Liste© [a drug formulary] was already known, filling in the ADR template also”* or *“already enough during studies”*). Not unexpected, paper cases are sometimes regarded as *“relatively dry”*.

#### 3.3. Student self-assessment

**Figure 1:**
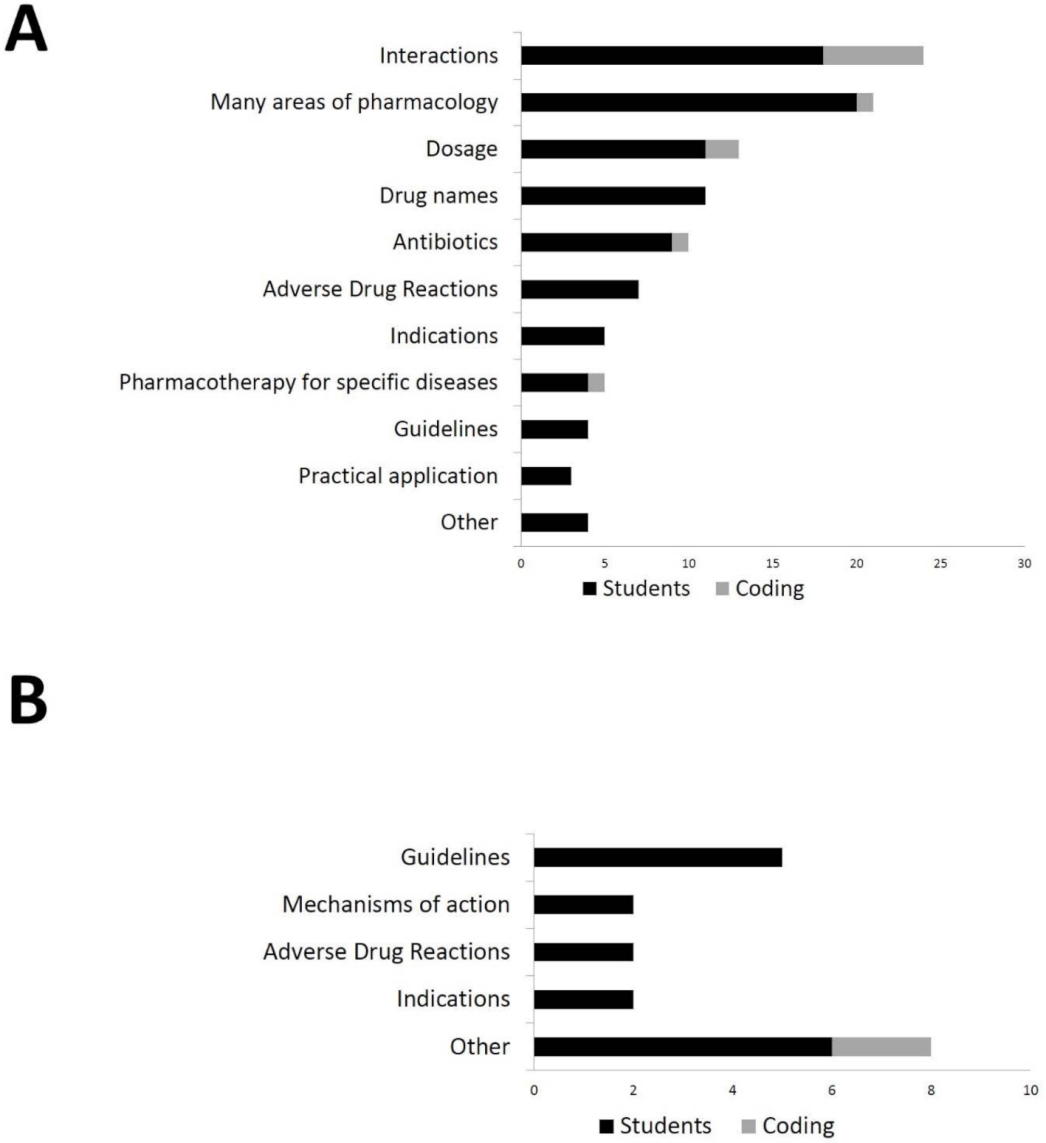
Respective number of in total 35 students (black bars) naming particular areas (subcategories from the content analysis) in interviews regarding perceived deficits (A) or learning successes (B). The frequency of denominations (= coding in the analysis of the interviews) of areas (grey bars) sometimes exceeds the number of students due to multiple mention in some interviews.

In the content analysis of the interviews, the main category “learning success” was divided into the middle categories “given” and “not given” (=deficits). Eleven of the 16 underlying subcategories were deficit categories (see figure 1 (Fig. 1), point A). In total, 107 statements of all 35 interviewees referred to areas of pharmacology and pharmacotherapy where students localized their own deficits. Only one subcategory referred to a specific group of drugs, namely antibiotics. Most frequently mentioned aspects were “interactions” (e.g.* “all those interactions with each other, I would always be uncertain about that”*), *“*dosages” (e.g. *“I’m not good at all at dosages”*), “drug names” (e.g. *“that one [...] cannot assign the drug names”) as well as *“adverse drug reactions” (e.g. lack of* “detailed knowledge about specific side effects”*). Many statements were unspecific and referred to uncertainty in many areas of pharmacology (e.g.* “not fit on any aspect of pharmacology”*). Nineteen statements of 15 students could be assigned to the middle category of achieved learning successes. Three of the five subcategories that have been derived from this (see figure 1 (Fig. 1), point B) were the same as for the deficits (“guidelines”, “indications” and “adverse effects”).

## 4. Discussion

The pharmacology modules of the PJ-STArT-Block focus on medication safety. Students are to sensitize to various relevant aspects of the medication process, e.g. to avoid medication errors and to help minimizing the potential risk of a medication therapy. An important step is becoming aware of risk factors and of sources of errors in order to develop preventive strategies [18], [19]. The participants of our PJ-STArT-Block modules find the discussion about handling and correcting medication errors positive and important. In addition, the evaluation shows that many students are becoming more aware of the importance of drug safety.

The interviews with PJ-STArT-Block graduates show that they perceive deficits in certain pharmacological areas. To some extent, these deficits are covered by the contents of our pharmacology modules, thus confirming their alignment. On the other hand, we cannot rule out the possibility that the students only became aware of certain deficits due to attending the modules. Studies indicate that one reason for errors in drug prescriptions is a lack of knowledge [20], [21], although final-year students’ self-assessment of knowledge and the quality of drug prescriptions show only a weak correlation [11]. An own study showed that the correlation between the proportion of correct answers in a multiple-choice test on pharmacological issues and the degree of certainty regarding the correctness of the answers was stronger among 9^th^ semester students than it was among those in their 6^th^ semester [22]. However, applying a statistical method for assessing the agreement between two methods of measurement (Bland-Altman-Plot) we found that the more advanced students tended to underestimate their overall level of knowledge. Given the model of decision quality proposed by Hunt it was more likely for 9^th^ semesters to apply correct knowledge, while 6^th^ semesters were more likely to hesitate or would even act amiss [23]. Thus, self-assessed deficits of PJ-STArT-Block graduates (which no doubt should be taken seriously) do not necessarily mean that lack of knowledge or uncertainty inevitably lead to medication errors. We do not know whether and to what extent students’ answers in the interviews were influenced by the experiences just made during the PJ-STArT-Block. However, a questionnaire survey conducted in the meantime shows no overall change in self-assessment during the course [17].

An essential element of the pharmacology modules is the physician-patient communication. A qualitative analysis of physician-patient conversations conducted here showed that 10^th^ semester students have considerable deficits in communicating a prescription [24]. Since the prescription talk plays an important role for medication adherence, among other things, this might be another relevant aspect regarding patient safety. The risk of medication errors due to lack of knowledge is likely to increase further if communication is inadequate. We previously found that medical students after participating in an elective course on prescription talks felt more confident and were more aware of the impact of physician-patient communication [25]. The simulations of physician-patient conversations in both the elective course and the PJ-STArT-Block probably revealed strengths and weaknesses regarding the integration of deciding on a drug treatment and communicating this to patients. This could then have enhanced awareness of the need for improvements in pharmacological education. A recent review [26] underlines that the simulation of patient conversations is particularly important in the field of pharmacology education. Students generally felt more confident about identifying, preventing, correcting and communicating medication errors. According to the authors, conducting conversations with simulated patients is furthermore motivating and increases awareness of patient safety. This is in good agreement with the evaluation results of our pharmacology modules of the PJ-STArT-Block.

It is plausible that formats such as those described here should be offered earlier during medical studies. Possibly, a realistic simulation would help to consolidate the knowledge to be acquired, similar to what was postulated for problem-based learning [27]. Though this would correspond to a recent recommendation of the European Society for Clinical Pharmacology and Pharmacotherapy (EACPT) [11], it should be noted that, depending on how advanced medical students are, it is not possible to come similarly close to reality. Regarding drug prescription, it should also be noted that so far only one teaching approach has been regarded as validated (“Guide to Good Prescribing” of the World Health Organization WHO [28], [29]). Though a review shows an advantage of simulation-based teaching over other formats, only a few of the included studies investigated medical students [26]. Interestingly, medical students in Germany prefer theoretical courses to practical courses in pharmacology, but this may be due to a lack of experience with the particular teaching formats [30].

## 5. Conclusion

The demand for a concerted approach to harmonization and modernization of teaching in clinical pharmacology and pharmacotherapy increases [11]. However, there is a lack of appropriate practice- and application-oriented teaching formats that go beyond the learning of declarative knowledge [11]. The PJ-STArT-Block at the University of Cologne is such an approach, for almost 10 years aiming to prepare students for their final practical year by giving the opportunity to face the upcoming medical requirements and responsibilities. In simulated physician-patient conversations about drug therapy, students become aware of deficits relevant for a safe drug therapy. Regarding drug safety, we consider the implementation of similar courses as necessary.

## Authors

The authors Verena Kirsch and Wencke Johannsen share the first authorship.

Wencke Johannsen is now employed in the pharmaceutical industry.

## Acknowledgement

EISBÄR and PJ-STArT-Block are educational projects of various institutions of the University of Cologne (Medical Faculty: Center for Palliative Medicine; Clinic and Polyclinic for Psychosomatics and Psychotherapy; Center for Pharmacology; Dean of Studies and Cologne Interprofessional Skills Lab and Simulation Center; Institute for History and Ethics of Medicine; Faculty of Human Sciences: Institute for Comparative Educational Research and Social Sciences). We thank Dr. Armin Koerfer for his support in content analysis. Thanks to Dr. Jessica Köth, Dr. Wiebke Seemann, Dr. Max Taubert and Dr. Martin Wiesen for their participation in the moderation of the described modules. Thanks to Dr. h.c. (RUS) Christoph Stosch for his consistent commitment to the continuation of the PJ-STArT-Block. EISBÄR and PJ-STArT-Block were funded by the Rectorate of the University of Cologne. Currently the PJ-STArT-Block is financed through means for quality improvement of the federal state of North Rhine-Westphalia.

## Competing interests

The authors declare that they have no competing interests. 

## Figures and Tables

**Table 1:**
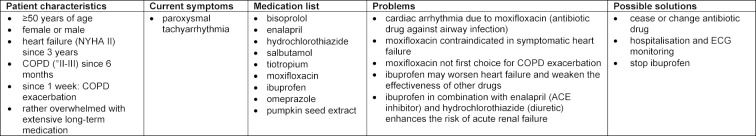
Module “Heart and Lung“ (S1) – Mrs./Mr. Reuther

**Table 2:**
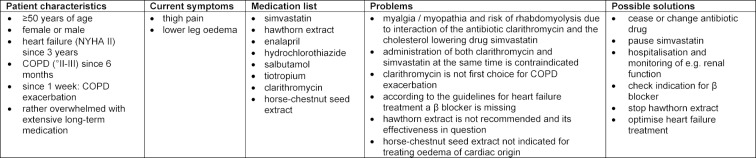
Module “Heart and Lung“ (S1‘) – Mrs./Mr. Oppermann

**Table 3:**
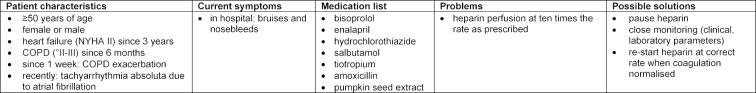
Module “Heart and Lung“ (S1‘‘) – Mrs./Mr. Steigmüller
